# On the Nature of Volition, Psychologically and Physiologically Considered

**Published:** 1862-10

**Authors:** J. Lockhart Clarke


### MEDICAL CRITIC

AND

### PSYCHOLOGICAL

### JOURNAL.

OCTOBER, 1862.

### Art. I.—ON THE NATURE OF VOLITION, PSYCHOLOGICALLY AND PHYSIOLOGICALLY CONSIDERED.

By J. Lockhart Clarke, F.R.S.

### Part I.

Perhaps nothing that relates to the moral and intellectual condition of man has been the occasion of warmer and more protracted discussion amongst philosophers than the nature of the *will*,—the nature of volition, and the question of *moral liberty*. The persistence of this celebrated controversy, and the tenacity with which the opposing disputants have continued to hold their opinions, may be doubtless attributed chiefly to the subtile, peculiar, and somewhat variable character of the actual phenomena to be considered. In the physical sciences, any given subject, as chemical affinity, heat, light, electricity, magnetism, &c., may be made to present exactly the same facts to the attention of every inquirer; for although the phenomena themselves are modifiable to a variable extent, according to the peculiar conditions of operation to which the forces that produce them may be subjected, still the same conditions, and consequently the same phenomena, can be always reproduced for the purposes of experiment or observation; so that any difference in the conclusions at which different investigators may arrive will depend almost entirely on a difference in their skill of manipulation, or in their powers of observation and reasoning. But in the department of moral science which we are now about to consider, the facts as experienced in consciousness for observation and analysis, are actually presented to each inquirer in a more or less modified form; for every one has a moral constitution peculiar to himself, partly as an original endowment, partly as it is modified by example and education, and dependent on the *strength* of the different passions, affections, and emotions, *in relation to each other and to the intellect*; so that by taking, as is frequently done, their own peculiar feelings or inward experiences as the subjects of inquiry, and generalizing the conclusions which they draw from them, that is, attributing to mankind in general what is attributable only to their own particular case, it is not surprising—nay, it must necessarily be, under such circumstances, that when men come to argue the question in the abstract, their judgments and conclusions will differ on certain identical points. Another cause of disagreement on the same subject may be traced to the powerful influence of religious faith and prejudice in biassing the faculties of the mind as instruments employed in the inquiry. Something, moreover, is due to ambiguity and misapplication of terms; but these are often the consequences of an inadequate conception and imperfect analysis of the facts or phenomena to which they are applied.

Let us, in the first place, inquire what is generally understood by a *voluntary* act. What is the *essential* element that distinguishes it from every other kind of act, according to almost every writer on the subject, both in ancient and modern times? "That which is voluntary," says Aristotle, "is found only in such (animals) as are *rational*."* Cicero, in expounding the doctrines of the Stoics, observes, "The will is that which desires with *reason*."† "The object of the will," says Hooker, "is that good which *reason* doth lead us to seek."‡ Sir Matthew Hale, after describing the understanding, observes that "the will is the other great faculty of the *reasonable* soul, and is not a bare appetitive power as that of the sensual appetite, but is a *rational* appetite."§ Dr. Erasmus Darwin gives a similar account of volition.|| Hobbes makes the Will to consist of the last appetite or passion in *deliberation*.¶ Condillac observes, "On entend

---

* Ἡ δὲ βουλευτική (φαντασία) εν τοῖς λογιστικοῖς.—*De Animâ*, lib. iii. cap. xi., also cap. ix. x., and *Rhet.*, lib. i. cap. x.

† Naturâ enim omnes ea, quæ bona videntur, sequuntur, fugiuntque contraria. Quemobrem simul objecta species cujuspiam est, quod bonum videatur, ad id adipiscendum impellit ipsa natura: id cùm constanter prudenterque sit, ejusmodi appetitionem Stoici βούλησιν appellant, nos appellamus Voluntatem; eam illi putant in solo esse sapiente, quam sic definiunt: *Voluntas est, quæ quid cum ratione desiderat.*"—*Tusculan-Quæst.*, lib. iv.

‡ *Laws of Ecclesiastical Polity*, book i.

§ *Origination of Mankind*, cap. 2, sect. i.

|| *Zoonomia*, vol. i. sect. xi.

¶ *Humane Nature*, chap. xii.

par volonté, un désir absolu, et tel que nous pensons qu'une chose désirée est en notre pouvoir."* "Volition," says Locke, "is an act of the mind *knowingly* exerting that dominion it takes itself to have over any part of the man."† Reid defines volition as "the determination of the mind to do or not to do something which we conceive to be in our power."‡ According to all these authorities, like many others that might be quoted, it is evident that *reason* or *intelligence* is the distinctive element of volition. The same conclusion is either explicitly understood by the majority of mankind, or implied in their intercourse with each other.

The term *voluntary* is derived from the Latin *volo, I wish, desire,* or *intend.* A voluntary action, therefore, is the result of an *intention, desire,* or *wish.* But every intention, desire, or wish has a certain intelligible object or end in view. We withdraw a limb from a painful position with the purpose or intention of removing the pain; but under the stimulus of acute suffering, the limb may be retracted through reflex action, without the concurrence of our wish or desire, and even in opposition to it; for although we may desire, for the sake of some future benefit, to keep the limb in the same painful position, yet all our efforts to do so may be overcome by the irresistible, and, therefore, involuntary tendency of the pain to produce reflex action. In the case of intelligent beings, therefore, all those actions which are performed without either the *guidance* or the *sanction* of intelligence, must be accounted involuntary; and conversely, none but a being capable of conceiving or realizing an intelligible object or end, can perform a *voluntary* act. By what term, then, should we distinguish all the actions of the lowest animals, in which there is no ground for believing that there is any conscious intelligence? They cannot, with the same propriety as the corresponding actions of intelligent beings, be called *in*voluntary; for this term should, strictly, be applied only to beings which are also capable of voluntary actions, that is, of actions guided by conscious intelligence. They are commonly, and, as would seem, properly distinguished by the term "instinctive." A purely *instinctive* action, then, is that of an animal which either does not possess any conscious intelligence at all, or whose intelligence contributes nothing to the action. In the first case it is always uncontrollable and involuntary; in the second it may be either uncontrollable or controllable, and therefore *in*voluntary or voluntary.

Spontaneous actions are those which originate from some prin-

---

* *Traité des Sensations*, chap. iii.

† *Hum. Understand.*, book ii. chap. xxi. ‡ *Active Powers,* essay ii. chap. i.

ciple or energy in the agent itself, and not from external mechanical force, or compulsion.* All *voluntary* actions, therefore, are spontaneous, but all spontaneous actions are not *voluntary*, since they are performed by the lowest animals which do not seem to be endowed with conscious intelligence; and even by some plants, which are devoid of every kind of consciousness. Such are the movements of the sponge-gemmules, the cells and zoosperms of the Protophyta, and parts of higher plants, resulting from the free operation of their vital forces. It is evident, therefore, that spontaneity, although one of the necessary conditions of volition, cannot constitute the Will. Spontaneity, indeed, is a mode of action of the vital or organic force, which may or may not have the additional attributes of consciousness essential to volition. In like manner, sensation, although in some form or other an element in volition, cannot alone constitute the will; for it is manifest that if animals were endowed with no other faculty besides sensation, every *tendency* to act, whether pleasurable or painful, would be uncontrollable, and *necessarily* result in action, unless it were checked by some other and stronger *sensation* excited from *without;* since, by hypothesis, there would be no other faculty within by which it could in any way be controlled. In such animals, therefore, there could be no *choice* or *preference* of one action to another, —a privilege which belongs exclusively to the will, and can be possessed only when the faculty of *intelligence* is superadded. In volition, then, there is either a distinct and explicit idea, or an immediate perception, of the *greatest* amount of *good* to be *obtained*, or of *evil* to be *avoided*, and the action which follows is the result of a comparison and intelligent selection, that is, of the animal's wish or will. One sensation is not, as in the unintelligent animal, immediately and directly superseded merely by another and stronger sensation; but mediately, through the faculty of intelligence, both are alternately compared together, or the idea of a past sensation is compared with one that is present. Actions, therefore, are accounted voluntary when the being which performs them understands and compares the particulars of the case, and acts with a choice and intention of his own.†

A writer of great ability on the nature of volition has recently

---

* Aristotle observes, Τὸ ἑκούσιον δόξειεν ἄν εἶναι, οὗ ἡ ἀρκὴ ἐν αὑτῳ.—*Ethic. Nich.*, lib. iii. c. i. And conversely, ἐοίκε δὴ τὸ βίαιον εἶναι, οὗ ἔξωθεν ἡ ἀρχὴ, μηδὲν συμβαλλομένου τοῦ βιασθέντος.—Ibid.

† Aristotle observes, Ὄντος δ' ἀκουσίου τοῦ βιαίου καὶ δι' ἄγνοιαν, τὸ ἑκούσιον δόξειεν ἄν εἶναι, οὗ ἡ ἀρχὴ ἐν αὑτῳ, είδότι τὰ καθ' ἕκαστα, εν οἷς ἡ πρᾶξις. *Since that which is involuntary is the result of force and ignorance, the voluntary would appear to be that of which the principle of action is in the agent himself, who at the same time knows the particulars in which the action is concerned.*—Aristotle: *Ethic. Nich.*, lib. iii. cap. i.

expressed an opinion which differs essentially from the one that I have just stated; for he maintains, and endeavours to prove at considerable length, that the only element necessary for rendering an action *voluntary* may be nothing else than sensation.* Every action, according to him, was at first spontaneous and involuntary, but acquires its voluntary character by an *accidental* association with some kind of sensation. His theory rests on the two following propositions: First,—From the moment of birth the different members of the body "begin to play of their own accord," in consequence of a store of nervous energy accumulated during nutrition and repose, and proceeding into action spontaneously, as an effusion of power of which the food is the stimulant.† Second,—"A pain increasing in company with a movement arrests that movement; and a pleasure increasing in the same connexion would stimulate and sustain that movement."‡

In these two laws of our constitution Mr. Bain considers that all the elements of volition are to be found. The first of these, or spontaneity of action, although "absolutely essential as a part, is certainly not the whole fact that we term volition. A second element is wanting for giving direction to those spontaneous workings, in order to invest them with the *character of purpose or aim*, belonging to the proper actions of the will."§ This second element consists of either pleasure or pain, as the "governing principle of our exerted might."|| "When there is a felt pain and also a movement spontaneously originated that happens to alleviate the pain, the spur to action of some kind, which is the property of a painful consciousness, urges the clutching and sustaining of that particular movement." According to Mr. Bain, it is this property or tendency of the pain to *sustain* the spontaneous movement that converts such movement into a voluntary action.¶ This is called by Mr. Bain an *inchoate* volition.** But "the *power of cohesion or association* operates to join together the two facts thus brought into *accidental* embrace, and after a time, the pain can stimulate the precise action, without any spontaneous coincidence of the two."†† This, Mr. Bain calls a "full-formed volition. Full-formed," he says, "because when the supposed pain can bring into play the proper movement, in the absence of all spontaneous tendency, we have a case of voluntary power complete for all the purposes of the living being."‡‡ It is evident, from various parts of his work, that under the term *pain* as a volitional element, the author

---

* Bain: *The Emotions and the Will.* 1859.

† *Emotions and the Will*, p. 330. ‡ Ibid. p. 344. § Ibid. p. 339. || Ibid. p. 343. ¶ Ibid. p. 343. ** Ibid. p. 353. †† Ibid. p. 343. ‡‡ Ibid. p. 353.

includes not only the pain associated with complex mental states of emotion, &c., but also simple organic pain or common sensation. Now Mr. Bain appears to have entirely overlooked the physiological fact, that pain both in its common and special form, not only may, but actually does give rise to movements, both reflex and direct, which are entirely beyond the control of any wish, desire, or effort *guided by the intellect,* and must therefore be accounted *in*voluntary. Such are the reflex movements of winking produced by contraction of the orbicularis muscle of the eye, under the stimulus of a strong and painful light, or some other kind of irritation; or the reflex actions of laughter from violent contraction of the expiratory muscle when excited through the medulla oblongata and spinal cord; or those of sneezing from irritation of the Schneiderian membrane of the nose. Such also, are some of the *direct centric* actions arising from a painful state of one of the nervous centres. In these cases, the *same pain* is as *immediately, invariably,* and *necessarily* followed by the *same muscular contractions* or movements, as the *same impression* is followed by the *same contractions* or movements in direct or reflex action *without* sensation. Whenever, therefore, a being acts from common sensation *alone,* he cannot act "with a purpose or aim, belonging to the proper action of the Will;"* for to act *with a purpose or aim* is to have the *choice* and the *power* of acting *in more than one way,* and to *choose one way rather than another;*—an act which can be performed only by a being which considers the circumstances of the case in question, that is, by a being which exercises its *intelligence* with the intention of performing certain actions and controlling others which would follow *necessarily* from sensation *alone.* And herein we perceive one of the final causes of the sense of pain—that when the reflex movements to which it immediately gives rise are of themselves insufficient to withdraw a limb or a part from the source of injury of which the pain is an indication, the feeling serves to stimulate the intellectual faculties to provide the means of accomplishing that end.

It is necessary, however, to observe, that Mr. Bain in a subsequent part of the same work, endeavours to prove that "sensation involves intellect," that is, that sensation is an intellectual process. Now before we proceed further, let us clearly understand what is here meant by sensation, and what is meant by intelligence or an intellectual process. Sensation, as here meant, is *physical* sensation, consisting of a painful or pleasurable, but simple, state of consciousness produced by the immediate or direct effect of an external *physical* impression on an organ of sense. By intelligence

* Bain, ut supra.

or an intellectual process is meant, *ideality*, or the *representation* of *past sensations* by *ideas; association* of such *ideas*, forming the basis of recollection or memory; and *comparison* and *judgment*, employed in determining their various *relations*. Now even the simplest of these intellectual operations, viz., the state of consciousness constituting an *idea*, is experienced as an unmistakeably *different kind* of consciousness from that constituting the actual *sensation* which it *represents:* the one kind, indeed, is incompatible with the other; for the moment we *think* of a sensation, or *represent* it as an *idea*, we cease to *realize* it *as a sensation*. Much more strikingly, then, is this incompatibility manifested between *numerous* or *complex sensations* and the *complex intellectual processes* of *analysis, comparison*, and *judgment*, by means of which their various *relations* are determined. In man, therefore, and the higher animals—of which alone we are now speaking—the *sensational* consciousness is psychologically distinct, although inseparable, from the *intellectual* consciousness.

In contending for the identity of *sense* and *intelligence*, even in man, Mr. Bain is far from being singular, for similar opinions were held by some of the ancient philosophers, and are maintained at the present day by several distinguished psychologists. "Sensation," says Aristotle, "is a certain kind of knowledge."* And again, "Sense, when separated from intelligence, has a kind of insensible office: hence the saying, 'intellect sees, intellect hears.'"† This last passage from Aristotle, which I have translated literally, is referred to by Sir William Hamilton (*Dissertation on Reid*, note D*). "There is extant," says Plutarch, "a discourse of Strabo Physicus demonstrating—*That a sensitive apprehension is wholly impossible without an act of intellect*."‡ Tertullian on the same subject observes, "Quid erit *sensus* nisi ejus rei quæ sentitur *intellectus*? Unde ista tormenta cruciandæ simplicitatis, et suspendendæ veritatis? Quis mihi exhibebit *sensum non intelligentem quod sentit*; aut *intellectum non sentientem quod intelligit*?"§ Now although the two faculties of *sense* and *intellect*, according to the definitions already given, always co-exist in the same being; although every sensation, in the normal state of the organism, excites an act of intelligence of some kind and degree; and although the first or original manifestation of intelligence was, in every being, dependent on sensation, yet these admissions

---

* Ἡ δ' αἴσθησις, γνῶσίς τις.—*De Animal. Generat.*, lib. i.

† Χωρισθεῖσα δὲ αἴσθησιν διανοίας, καθάπερ ἀναίσθητον πόνον ἔχει, ὥσπερ εἴρηται τὸ, νοῦς ορᾳ, καὶ νοῦς ἀκούει.—Aristotle: *Prob.* xi. 33. See also *Analyt. Post.* lib. ii. last chap.; *Topica*, lib. ii. cap. 4; and *De Animâ*, lib. iii.

‡ Plutarch: *Op. Mor.* *Hamilton's Reid.*

§ *De Animâ*, c. 18. Compare *De Carne Christi*, c. 12; also St. Gregory: *De Opif. Hom.*, cc. 6, 10; and *De Animâ et Resur.* *Hamilton's Reid*, Note D*.

are very far from implying that there is no actual difference in kind between *sense* and *intellect;* or that their *co-existence* in the same being constitutes their *identity.* If, according to Tertullian, Sir William Hamilton, and other modern philosophers, to *feel as common sensation* is to *understand* what is *felt;* and to *understand* is only to *feel as sensation* what is *understood;* then we must accept all, and more than all, that is contained in the sensational system of Condillac and Cabanis. "But who," says Tertullian in the passage above quoted, "will show me *sensation* which does not *understand* that which it *feels;* or *intelligence* which does not feel what it understands?" Now if between the two states of consciousness there be any difference at all corresponding to the difference between the terms employed to express them, then, to say that we can be conscious *as sensation* only *by intelligence,* and be conscious *as intelligence* only *by sensation,* is to assert that the two states are *reciprocally* and equally dependent on each other for their actual *origin* and continuance—an assertion which amounts to a plain contradiction and absurdity; for if sensation by the senses and understanding by the intellect be two distinct states, which are chronologically incompatible with each other, it would follow from the same assertion that we must *feel* before we can *understand,* and yet *understand* before we can *feel.* To *understand* a sensation is not actually to *feel* it, but to *recognise* it by an act of *memory* or *association,* as similar to sensations or a sensation *formerly felt,* and refer it to the same class. Tertullian, indeed, appears to have confounded *co-existence* with *identity.* We know by an act of intelligence (memory) that we are experiencing or have just experienced a sensation of a definite kind; but the *idea* or *thought* of that sensation, which is another and distinct state of consciousness, succeeds it so rapidly, and is often so vivid, that we are apt to judge of the two states as *co-existing* at *one and the same instant of time,* and as identical in kind, especially when they are *repeated alternately* in rapid succession. If Tertullian had asked, "Who will show me the man that does not *understand* the *kind* of sensation which he feels or has just felt?" he would have proposed a very reasonable question.

But as the subject is one of considerable importance, let us examine the arguments brought forward by modern writers in support of the opinion that no real distinction can be drawn between sensation and intelligence, as they occur in man and the higher animals, according to the definitions I have already given. To begin with Sir William Hamilton. After enumerating the conditions of *Perception,* amongst which he includes *attention, memory, abstraction,* and *judgment,* he continues, "Such being the general *conditions* of *Perception,* it is manifestly impossible to discriminate with any rigour *sense* from intelligence."* But singular enough, Sir William Hamilton, who understood the nature of *Sensation* proper, has here *identified* it with *Perception* proper, which, as is evidently implied in some other passages of his writings, is distinguished from *sensation* by those very conditions above mentioned, which constitute *intelligence*, viz., attention, memory, abstraction, and judgment. "Perception proper," he says, "is the consciousness, through the senses, of the *qualities of an object known as different from self.* Sensation proper is the consciousness of the *subjective affection of pleasure or pain*, which *accompanies* that *act of* knowledge. Perception is thus the *objective element* in the complex state—*the element of cognition;* sensation is thus the *subjective element*—the *element of feeling*."† And in another place he draws a decided line of distinction between sensation and intellect, and even opposes the one to the other. "In the phenomena of Feeling," he says, "the phenomena of Pleasure and Pain—consciousness does not place the mental modification or state before itself; it does not contemplate it apart—as separate from itself—but is as it were fused into one. The peculiarity of feeling therefore is, that there is *nothing* but what is *subjectively subjective;* there is *no object different from self*—no objectification of any mode of self. We are indeed able to constitute our states of *pain and pleasure* into *objects of reflection*, but *in so far as they are objects of reflection, they are not feelings*, but only *reflex cognitions* of feelings."‡ Now if in Feeling or Sensation there be nothing but what is "subjectively subjective," and feelings or sensations cease to be such so soon as they become objects of reflection or intellect, as asserted in this passage, it is impossible, as the author elsewhere states, that "*an act of intelligence* is to be viewed as the *principal factor in the percipient process, even in its lower form, that of sensation proper;*"§ and that "*Sensitive apprehension* is, in truth, only the recognition *by intelligence* of the phenomena presented in and through its organs."|| It is true that in man and the higher animals no sensation can be experienced without exciting some act or process of intelligence, although such an act cannot excite sensation, the objects of which must be presented through the organs of sense; but whenever these objects *are* presented to the senses, the *feeling* or *sensation* is not a process of intelligence, or, as Sir William Hamilton asserts, "a recognition *by intelligence* of the phenomena presented in and through its organs;" although it *is* a recognition of the phenomena through its organs by a being which, besides

---

* *Dissertations on Reid*, Note D*, 11. † *Lectures on Metaphysics*, vol. ii. p. 99.
‡ *Lectures on Metaphysics*, vol. ii. lect. xlii.
§ *Dissertations on Reid*, Note D*, 22. || Ibid. 11.

the capacity of *sense*, is endowed with the capacity of *intelligence*. But so long as the consciousness assumes the one state it cannot exist in the other.* The truth seems to be that in man and the higher animals, the conscious entity or principle is endowed with different properties, or capable of existing in different states, each of which, however, can be manifested only through its own special and material organ. All that we can recognise by *intelligence alone* is the idea of a sensation, or the *relations* between such ideas, which we are conscious of, not *as sensations*, but as their *ideal re*presentations which we can experience only when the sensations are *past*. Moreover, in involuntary actions accompanied with sensation, such as coughing, sneezing, vomiting, we are conscious that our intellect contributes nothing to the movement, but is often exerted to oppose it; while we always know when we assist or imitate such movements by an *intelligent* act of volition.

The ground on which Sir William Hamilton supports his opinion is, that the mind as *sensation* cannot feel by being merely a "passive affection of the organism:" "it can be said to feel only in being conscious of itself as purely active:" and this activity, according to him, it can derive only from *intelligence*, as *a concentration* and judgment of the mind "spontaneously apprehensive of an object-object, or mode of the non-ego, and not of a subject-object, or affection of the ego."† This activity, then, is *attention* (a concentration of the mind upon an object) and judgment. But the activity of *attention* is the *re*-action of the mind upon a *previous* action made upon it through the senses and experienced as *sensation*. The sensation is the *first conscious effect* of the external impression *on the mind*, and is received *involuntarily*. The *attention* is the subsequent *reaction or reflection of* the mind upon the impression on the organ of sense, in consequence of the *sensation* possessing more interest than any other sensation experienced at the same moment, and is a *voluntary* act. The involuntary effect, then, of the external impression experienced as *sensation* is the necessary antecedent and condition of the reflex activity of attention and judgment.‡ But we know

---

* This statement would be true even if the same material organ or part of the brain were subservient to both these states of consciousness.

† *Dissertations on Reid*, Note D*, pp. 859 and 881. See also Note D*, 14, 15, and 16.

‡ The late Mr. James Mill endeavoured to show that there is no difference between *sensation* and *attention*. (*Analysis of the Phenomena of the Human Mind*, vol. ii. A work displaying great acuteness and logical precision.) It is well known that Condillac derived all the faculties of the mind from *sense*, contending that all the intellectual processes are mere modifications or transformations of *sensation*, or to sum them up in his own words, "que la sensation enveloppe toutes les facultés de l'ame."—*Traité des Sensations*, chap. vii.

Cabanis, a follower of Condillac, starting from the *Traité des Sensations*, directed that an impression made on a nerve-centre, even when unattended by sensation, is not a merely passive effect; for within that centre it causes the production of new nerve-force which always *re*acts in some direction or other; so that we have here an instance of a new development of force, or activity, which is not only not derived from *intelligence*, but not even from *sensation*. And a similar production and reaction of new force take place, as the *immediate effect of sensation*, or rather of excitement of the nerve-centre through which sensation is experienced. It is evident, therefore, that sensation cannot be merely a "passive affection of the organism." And to say that the mind, *considered as a separate entity*, cannot *feel*, by *being* merely a "passive affection of the organism,"—if it has any meaning at all—which is far from evident,—can mean nothing else than that the mind, as *a separate entity*, cannot feel *so long* as the effect of the external impression is *confined* to the *material organism*,—which is clear enough, and the very point for which I contend, viz., that the mind, as a separate entity, must receive the impression from the brain, and be *acted on* or *excited* by that impression before it can be conscious of its existence; and must be conscious of its existence before it can *react* as *attention*.* When we experience some *in-*

---

his attention chiefly to the source and physiological relations of Sensation, as manifested through the nervous system or its material organs.—*Rapports du Physique et du Moral.* He might be called the physiologist of the sensational school.

Destutt de Tracy, while he accepted the physiological doctrines of Cabanis, limited his inquiries to the psychological relations of the subject, and endeavoured, by a series of subtle and exact analyses, to trace the conditions under which the different faculties of the mind are successively developed from sensation.—*Eléments d'Idéologie.*

In Laromiguière, the sensational school encountered a strenuous opponent. He denied that sensation is the only element, or the starting-point, of all the other faculties of the mind. Those faculties, he contended, can be derived from *sensation* in so far only as *attention* is derived from it. But *sensation*, he continues, is *passive*, while *attention* is *active*. Attention, therefore, is not derived from sensation. The difference between the two is shown in the different meaning of the words which are used to denote their actions, and is perhaps shown better in the French. "Partout," says the author, "on *voit* et l'on *regarde ;* on *entend* et l'on *écoute ;* on *sent* et l'on *flaire ;* on *goûte* et l'on *savoure*."—*Leçons de Philosophie.* See also Cousin : *Fragmens Philosophiques.* 1826, p. 20.

There appears to be really no essential difference between the modern sensational doctrine and that held in ancient times by Protagoras and others, who maintained that whatever any one *knows*, he knows through *sense*, or feels ; and that *science* or knowledge is nothing but *sense*. *Δοκεῖ οὖν μοι ὁ ἐπιστάμενός τί αἰσθάνεσθαι τοῦτο ὅ ἐπίσταται.——Οὐκ ἄλλο τί ἐστιν ἐπιστήμη ἢ αἴσθησις.*—Plato : *Theætetus*, in which the doctrine is fully examined and opposed. This quotation is equivalent to the remark of Destutt de Tracy, that "penser c'est sentir." For a critical examination of Protagoras's doctrine, see also Cudworth's fine treatise on *Eternal and Immutable Morality*, book ii. chaps. v. and vi. and book iii.

* We shall hereafter see that many distinguished writers, particularly Dr. Samuel Clarke and Victor Cousin, have held an opinion entirely opposite to that of Sir W. Hamilton, with regard to Intelligence, by denying to it *all Activity*, and for that very reason all participation in the beginning of motion, and therefore in acts of the will or volition.

voluntary movement excited directly by *sensation*, such as spasmodic coughing, sneezing, or painful closure of the eyelids, which we desire and endeavour with all our energy, by an act of volition, *to prevent*, we are certainly conscious that the *whole activity* of our minds, as *intelligence*, is *concentrated in the effort to oppose* those movements, and therefore, *as intelligence* cannot be the dynamic or active principle of the sensation from which they spring, but on the contrary must be directly opposed to it. Hence it is that such actions are considered *in*voluntary and not our own actions, because they are neither begun nor directed by that principle of our nature, the intellectual, which is necessary to constitute an explicit desire or wish, and which is the distinctive element in our characters as personal and responsible agents. When my consciousness exists solely as intelligence, that is, when it is wholly occupied in thought upon some other subject, and a sharp instrument *unexpectedly* pierces my skin, the first intimation I receive of the presence of that instrument is through the sensation of pain which it inflicts, but which I am certainly not conscious of *feeling* by a process of thought or intelligence; for although my mind be diverted from its previous object and be now occupied with the *sensation*, yet *so long* and *so far* as it is so occupied, it must, as already shown, cease to exercise any act of intelligence. Again, when some irritating particle enters my eye or touches my larynx, I *feel* not only that the pain is quite independent of, or prior to, any exercise of intelligence on my part, but also that the reflex movements which immediately ensue may be in direct opposition to my desire and the dictates of my intellect; although that intellect, when it sanctions or coincides with those movements, may assist or prolong them by a voluntary effort, and even imitate them when the pain or sensation is absent.

Let us now examine the arguments by which Mr. Bain considers himself justified in concluding that "sensation involves intellect." "*All* sensations," he says, "after the *first* of each kind, involve a flash of *recovery from the past*, which is what really *determines their character*. The *present* shock (of feeling) is *simply made use of as a means of reviving* some one *past* in preference to all others."* Now by a careful examination we shall find that each of these sentences refutes, by implication, the very proposition which it is intended to confirm; for in the former it is clearly implied that the *first* sensation does *not* involve *a recovery from the past*, and therefore shows an instance of *sensation* that does *not* involve *intellect*, since *a recovery from the past* is an act of remembrance, or intellect. Again, every sensation

---

* *Emotions and the Will*, p. 631.

must be the consciousness of the *present* moment *as sensation;* otherwise it would not be a sensation, but the *idea* of a sensation: therefore no true sensation *can* involve *in itself* a flash of recovery from the *past;* for such recovery being an act of remembrance, and existing as an *idea,* must, just in that proportion, supersede the *present* shock or sensation, which cannot itself remember, but, "*as a means* of reviving" the *past,* "*is simply made use of*" by the mind, *now existing* as an act of *intelligence,* or as a state of consciousness in which the properties of both sensations are identified, but which succeeds the last so rapidly as to appear identical with it. It follows, then, that in man and the higher animals sensation can and does actually exist as a state of consciousness quite independent of, although inseparable from, intelligence, which, however, in the normal state, it always excites or passes into. Sir William Hamilton himself has announced the law, "*That perception and sensation* in the degree or intensity of their existence, are always found in an inverse ratio to each other."* In all cases in which, amongst the higher animals, sensation *appears* to "involve" or possess the properties of intellect, the two states succeed each other, either simply or alternately, with such extreme rapidity, that their difference escapes notice. Even if, as Mr. Bain believes, the cerebral hemispheres and the same particles of those hemispheres minister to both sensation and intellect, the identical nature of these states would not thereby be proved, since they are chronologically incompatible with each other and must follow alternately or in rapid succession; and there would be as much reason for distinguishing the one from the other, as there is for distinguishing *pleasurable* from *painful* sensations, which are also *alternate*

---

* *Dissertations on Reid,* Note D*. In some of his foot notes, Sir William, partly by implication, and partly by direct statement, draws between sensation and intelligence the distinction which he elsewhere denies. "The perception," he says, "of parts out of parts (that is *extension*) is not given in the *mere affection of colour,* but is obtained by a *reaction* of the mind *upon such affection.*" (Note D, 18.) Here the sensation (affection of colour) is clearly distinguished from *attention,* or the "*reaction* of the mind *upon such affection*:" which two states are elsewhere identified by Sir William, as already shown. (See § above.) "Again," Sir William says, "if we take a survey of the senses, we shall find, that exactly in proportion as each affords an idiopathic sensation more or less capable of being carried to an extreme either of pleasure or pain, does it afford, but in an inverse ratio, the condition of an objective perception more or less distinct. To a dog in whom the *sense* of smell is so *acute,* all odours seem, in themselves, to be *indifferent.*" Note D, 22. Now, according to this statement, it is evident that the *intensity* of *sense* is in the *inverse* ratio of the *power of discrimination.* But this discrimination is a judgment of the mind, or an act of intelligence: therefore the *intensity* of *sense* in the *inverse* ratio of intelligence. We have seen, however, that, according to Sir William Hamilton, *sensation* is only *possible* as an act of intelligence,—"as a *discrimination and judgment of the mind;*" therefore the *intensity of sensation* is both in the *inverse* and *direct* ratio of discrimination and intelligence, which is absurd.

and *different* states of consciousness dependent on *different* and *alternate* affections of the *same* nerve.

Mr. Morell is another of the modern psychologists who can discover no difference between sense and intelligence. In corroboration of his opinion he quotes from Sir William Hamilton one of the passages to which I have referred. "What we have to consider," he continues, "under the term sensation, is mind,—the *entire* mind, on the lowest, and, if we may so express it, the most physical stage of its activity."* Now, looking at the mind as an entity endowed with a variety of properties, or capable of existing in a variety of states, it may be allowed to be *entirely* present, or engaged in *sensation,* if we admit that in man and the higher animals each of its conscious states can exist only successively, and must depend on a special material organ for its manifestation; for then so long as the entire mind is affected only by its *organs of sense,* the conscious state will be that of sensation; which, however, may instantly pass away to be succeeded by intelligence when the same entire mind is influenced through the cerebral organ upon which that state of consciousness depends. So that, although, in any one animal, the *entire* mind may for a time be manifested as sensation, yet sensation is only one of its conscious states wholly distinct from the others, which exist indeed *potentially* at the same moment, but can be *actually* manifested only, and that successively, through their respective material organs. The error consists in supposing that because the *faculty,* or (as Mr. Morell dislikes that term) the capability of *intelligence* always co-exists in the same being with the capability of *sensation,* the two conscious states must be identical in nature, or that sensation is nothing but intelligence. Some of Mr. Morell's subsequent remarks are at variance with the doctrine which he supports. "Sensation proper," he says, "in relation to knowledge, is *wholly subjective.*" Speaking of *shuddering, twinging, cramps,* &c., he observes: "Here the process stops, as far as the *sensation* is concerned; for any inquiry into the cause or into the object of such feelings would be an *intellectual* process *clearly distinguishable* from the sensation itself.† How can we reconcile this with the statement which Mr. Morell quotes from Sir William Hamilton,—"That it is manifestly impossible to discriminate with any rigour Sense from Intelligence?"

Mr. Mansel is the only other metaphysician that I shall notice as holding similar opinions with regard to Sense and Intelligence. His statements, like those of most other writers in support of that doctrine, are certainly contradictory, and therefore self-destructive. "Let us," he says, "suppose the existence of

* *Elements of Psychology,* p. 86.

† *Op. Cit.* p. 112.

a being furnished with human organs of sensation, but with no power of remembering or reflecting upon the objects presented to them." That is, we are to suppose a being endowed with the capacity of *sensation*, but without *any intelligence whatever*. "It is possible," continues Mr. Mansel, "that in such a case, though diverse objects might be successively presented to the senses, yet there would be no consciousness of their diversity; for such consciousness requires the juxtaposition of the objects in the mind, and this can only be effected by memory." (Which memory, being an act of intelligence, is of course *absent* in the case supposed.) "His consciousness," continues Mr. Mansel, "if consciousness it could be called, would probably be no more than an indefinite sense of uneasiness, a feeling of momentary irritation in the organ affected, but without discerning in what manner it is affected, and without distinguishing the permanent self from its momentary affection."* So far the author's assertions are consistent, and his conclusions appear to be correct. But this state of simple sensation, or purely subjective feeling, which by hypothesis is wholly unable to perform the *simplest* and most rudimentary act of intelligence—that is, to connect itself with, or recall, any *past* experience—this wholly unintelligent state of feeling is pronounced by Mr. Mansel to be "the *lowest degree of intelligence*," the germ of consciousness, but not itself entitled to that name; as being deficient in the essential conditions of limitation and difference, not having realized the distinction between subject and object, or between one object and another."† In these two passages, then, we have the plainest contradiction in the assertion that sensation, even as it exists in man, is essentially both *different from* and *identical with* intelligence! Now even if there could be found a case like that supposed by Mr. Mansel,—that is, a being furnished with human organs of sensation but without any capacity of intelligence, such a being at the *instant* the sensation was excited would probably experience the same kind of consciousness as another being endowed with a capacity of the highest intelligence would feel at the first moment of life, before that intelligence had reacted and rendered its subjective sensations definite and distinct.‡

---

* *Metaphysics*, pp. 41, 42. † Ibid. p. 42.

‡ Of the influence of intelligence in rendering our *subjective* sensations *objective* or definite and distinct, we have examples in the acquired perceptions of sight, hearing, and touch. During these acquisitions, however, there is no exercise of abstract thought—there is no formation of general propositions that are applicable to all other cases of the same nature. The process, indeed, is carried on by induction and association; but the reasoning of the infant mind is applied only, or limited, to the individual facts immediately before it. Hence it is that in after years the steps of the process are unknown, as they were not themselves made separate *objects* of thought. In the mature state of the human mind, however, some

Although through the intellect we may form and retain a *permanent* and vivid idea of the "momentary irritation" which excites it, we cannot prolong the irritation or *sensation* in the absence of the objects which caused it; and the exercise of the intellect in distinguishing the "*permanent* self" from its "momentary affections" does not in any way change the nature of that affection, or render it less *real*. Sensations must be *felt*, or *experienced as such*, before they can be *analysed, compared*, or *considered* in their relations to each other. They must therefore be *prior* to intelligence, and thus far independent of, although inseparable from it. So that "the distinction between subject and object, or between one object and another," is an intellectual process excited by, and therefore subsequent to the sensations, upon which it must consequently depend for the very conditions of its performance.

It is true that when we descend to the lower classes of animals —to the articulata, for instance,—there can be no actual distinction drawn between sensation and intelligence. There is probably no state of consciousness experienced as intelligence apart from sensation, but both are, as it were, fused into one, or into a state of indifference, constituting that which we call *Instinct*. In this, whatever sensations are experienced, must be very different from the distinct and definite sensations of the mature human being; and whatever apparent intelligence may be displayed must be attended with a very different kind of consciousness from that which belongs to the distinct and definite form of intelligence in man.* The peculiarity of instinct appears to consist in the

---

act of abstract thought appears to be necessary for the full and perfect exercise of the senses, as Mr. Mansel has shown in another and valuable work on *The Psychological Character of Logical Processes*. "In mere intuition," he observes, "all that is simultaneously presented to the sense appears as one whole; but mere intuition does not distinguish its several parts from each other under this or that notion. I may see at once, in a single panorama, a ship upon the sea, an island lying behind it, and the sky above it. To mere intuition this is presented only in confusion as a single object. To distinguish its constituent portions, as sea and land, ship and sky, requires a comparison and classification of them relatively to so many separate concepts existing in the mind; and such classification is an act of thought."—*Prolegomena Logica*, pp. 10, 11. With regard to the exercise of intelligence on our originally subjective feelings, it is also well remarked by a very profound, clear, and comprehensive thinker, "that during the first stage of incipient intelligence, before the feelings produced by intercourse with the outer world have been put in order, there *are* no *cognitions* strictly so called; and that, as every infant mind shows us, these slowly emerge out of the confusion of unfolding consciousness as fast as the most frequently repeated sensations, and their relations to each other become familiar enough to admit of their recognition as such or such, whenever they recur."—Herbert Spencer: *System of Philosophy. First Principles*, part i. p. 80.

* Aristotle has observed that in man the sense of smell is always either grateful or painful; that animals with hard eyes (*τὰ σκληρόφθαλμα*) probably perceive colours in a similar way, and that differences of colour impress them with a sensation of fear, or otherwise.—*De Animâ*, lib. ii. c. ix.

entire absence of any *conscious* end or design. The organic changes in the nervous centre which constitutes its material organ, while they are attended with some kind of consciousness, and carried on according to the laws of intelligence, conformably with the special requirements of each individual organism, appear necessarily to expend their force *at once* on the motor organs, which are not only adapted to respond, but are suitably constructed for performing the actions and securing the ends for which they were designed by the Creator. In man, however, the organic changes which occur in the sensational and instinctive centres are *not* necessarily expended *directly* on the instruments of motion, but are first succeeded by another series of similar changes which they excite in a special organ of intellect, through which their effects are either controlled, modified, or guided for the attainment of special and *conscious* ends. Hence it is that the operations of pure instinct are said to be blind, uncontrollable, and therefore *involuntary*. It has been well observed by Mr. Morel, "That so long as intelligence takes the form of a mere subjective experience, nothing can become, in the logical sense, definitely known or clearly comprehended. Before an object can be distinctly understood, it must first be projected out of ourselves, made to stand altogether apart from our immediate experience, and assume the character of an independent intellectual reality. Until this is the case, all intelligence is, if we may so express it, in a *fluid* state; it comes to no shape, crystallizes into no clear conceptions, but remains wholly identified with the momentary feelings of the thinking subject."* But although in the lowest animals there is this apparent identity of sense and intelligence, which seem as it were to be fused into one common state of consciousness, yet when we find them, in the course of development, either in the fœtus or in the scale of animal life, emerge each in a distinct and different form, out of that common or indifferent state, are we to ignore the distinction, and assert with Sir William Hamilton and others, that sensation is simply a *function* of intellect? It might with equal reason be maintained that there is no real difference between the brain and the foot, or between any other two organs of the body, because in the ovum they are both developed out of one homogeneous tissue, or common germinal mass.†

---

* *Elements of Psychology*, pp. 165, 16.

† The *cephalic ganglia* of the articulata are sometimes regarded by physiologists as homologous with the *corpora quadrigemina* of the vertebrata. This opinion appears to me entirely erroneous. The *corpora quadrigemina* are subservient to only *one* of the senses—the special sense of vision; and are normally the *direct* centres of only a *small number* of muscular movements of a uniform and purely reflex character, excited through the eye by impressions from *without*. On the other hand, the *cephalic ganglia* of articulata are not only the centres of vision, but

Let us next consider the manner in which we acquire voluntary control over our muscles, or the power of directing and co-ordinating their actions for special and intentional ends. According to Mr. Bain, we never could have succeeded in learning how to perform any one voluntary movement, unless that particular movement had already occurred *spontaneously* at the same moment with some *accidental* pain which it *happens* to alleviate, or with some *accidental* pleasure which "urges the clutching and sustaining" of it; so that all our voluntary acquisitions consist of a multitude of couples of individual sensations and individual movements, joined by association, after being commenced by spontaneity."* In this theory, however, there is nothing new; it is included in that which Hartley based on the *law of association,* and which Priestley adopted and defended.† Hartley divided muscular movements into four classes. 1. *Automatic;* 2. *Semivoluntary;* 3. *Voluntary;* and, 4. *Secondarily Automatic.*‡ All muscular motions were originally *automatic.* By association, however, they become connected with other *motions,* with *sensations,* with *ideas,* and with *emotions* or

---

the common and co-ordinating centres of all the other senses, when several exist in the same being. Moreover, they are the common centres of different *instinctive* feelings, impulses, or appetites excited by different conditions of certain *internal* organs, and operating according to the laws of intelligence, but without any *conscious* design, co-ordinate in different ways, at different times, under the guidance of the senses, *all* the muscular movements of the body. The *cephalic ganglia,* therefore, *in a general way,* bear the same kind of relation to the organism of the articulata as the *whole* encephalon or brain bears to the organism of the higher animal. But in the encephalon of the latter a distinct nerve-centre presides over each of the more highly-developed organs of sense, while the separate functions of intellect are entrusted to a special cerebral centre.

* *The Emotions and the Will,* p. 353. Müller regarded even the automatic movements of the fœtus as voluntary. (*Physiology,* vol. ii. p. 935.) Mr. Bain, however, in another of his works, observes that "Müller's application of the term *voluntary* to the initial movements, prompted solely by the state of tension of the nerve-centres, is not strictly correct; these movements are but one term of the couples that make up an act of volition, both a feeling and a movement are necessary parts of every such act." *The Senses and the Intellect,* p. 292.

† Sir James Mackintosh attributes the discovery of the *law of association of ideas* to Hobbes, and remarks that "its full application to the whole intellectual system we owe to Hartley, who stood in the same relation to Hobbes as Newton to Kepler; the law of association being that to the mind which gravitation is to matter." Long before Hobbes, however, this law was known to many other writers, who had it from Aristotle, as Coleridge has shown in his *Biographia Literaria,* vol. i. chap. v.

‡ Hartley had a very clear conception of *reflex action,* as is evident from the following passage:—"We are to conceive," he says, "that these sensory vibrations which are excited in the external organs, and ascend towards the brain, when they arrive in their ascent at the origins of motory nerves, as they arise from the same common trunk, plexus, or ganglion, with the sensory ones affected, detach a part of themselves at each of these origins down the motory nerves; which part, by agitating the small particles of the muscular fibres, excites them to contraction." (*Observations on Man,* Proposition 18.) In this way he accounts for the actions of sneezing, swallowing, coughing, hiccoughing, vomiting, and defæcation. Prop. 19.

*desires*, so as to be excited no longer in the *automatic* manner, but merely by the previous introduction of the *associated motion, sensation*, or *idea*. Sensations and ideas become connected with each other by association in the same way. Hence the whole doctrine of association may be comprised in the following theorem :—

" *If any sensation* A, *idea* B, *or muscular motion* C, *be associated for a sufficient number of times with any other sensation* D, *idea* E, *or muscular motion* F, *it will, at last, excite* D, *the simple idea belonging to the sensation* D, *the very idea* E, *and the very muscular motion* F."*

By this law of association, motions *originally automatic* become *voluntary*. Thus the automatic action of grasping, in the young child, is associated in the brain with the visible appearance of a favourite toy which the child uses to grasp, so that he repeats the act of grasping when the toy is again presented to his sight. After a sufficient repetition of the proper associations, the sound of the words *grasp*, *take hold*, &c., the *sight* of the nurse's hand in a state of contraction, the *idea* of a hand, and particularly of his own in that state, with innumerable other associated circumstances, *i.e.*, *sensations, ideas*, and *motions*, will put the child upon grasping, till at last, that idea or state of mind which we may call the *will* to grasp is generated.†

Such is a very brief statement of Hartley's theory of voluntary control over muscular movements. It differs from that of Mr. Bain in not considering those movements as voluntary which are excited by *sensation alone* ;‡ but still too much is attributed to *accident* and *accidental* association. That this *accidental* association is the means by which a great number of movements necessary for the alleviation of suffering and the procuring of enjoyment are originally discovered by the infant, there can be no doubt; but that this is the *only* means,—that *all* such movements, in fact, and still more, that every kind of voluntary action must, as Mr. Bain contends, " wait upon the *accidents* and improve them when they come," so that *without these accidents* " voluntary control could not find a starting-point," appears to be entirely opposed to what may be observed and learned by every day's experience; for while, with the express design or intention of relieving pain or obtaining pleasure, we try, and teach ourselves, to perform a number of voluntary movements which *never were* spontaneously and *accidentally* associated with such pleasure or such pain, there is an infinite number of other move-

* *Observations*, Prop. 20. † Prop. 21.

‡ " Of the two sorts of motion, viz., automatic and voluntary, the first depends upon *sensation*, the last upon *ideas*." Hartley, *Observations*, Prop. 15.

ments that have no immediate connexion with physical pleasure or pain, but are expressly intended to be subservient to the endless variety of desires that are excited by the wants, tastes, or ideas constantly arising in the course of our daily avocations and transactions in life, and which frequently require, in accordance with the end in view, such a peculiar and complex combination or co-ordination of muscles as never could have occurred accidentally, and which nothing but repeated trials could possibly accomplish. According to Mr. Bain, even the actions of eating, drinking, sucking, smelling, &c., both in man and the lower animals, are learned by experience, and "first hit upon in a course of *groping* and *accident*."* Hartley, however, regarded the actions of suction, mastication, and deglutition as originally automatic and *reflex*, and dependent on sensations of the lips, tongue, and fauces, but capable of being improved and rendered voluntary by association.† But Priestley, like Mr. Bain, regarded the act of sucking as a voluntary acquisition from the first.‡ "The action of sucking," he says, "I am confident, from my own observations, is not natural, but acquired; and so I believe are all the actions which Dr. Reid and others, who judge superficially in these cases, refer to instinct." But while it must be allowed that Reid multiplied instinctive actions to a great and unnecessary extent, Priestley appears to have run into the opposite extreme, by denying their existence altogether, and attributing everything to *accident* and *experience*. It appears, *à priori*, reasonable to expect that the performance of an action like that of sucking, upon which the urgent wants of the infant are immediately dependent, would not have to wait for the teachings of experience; and correct observation, as well as physiological facts, attest the soundness of this conclusion. To regard the act of sucking as an acquired process, reducible to the law of *accidental* association of sensation and motion, is as incorrect as it would be to account in the same way for the actions of breathing, or crying, or for those automatic or spontaneous, but, at the same time, harmoniously co-ordinate actions of flexion, extension, &c., upon which these philosophers allow that voluntary acquisition must originally depend. In man and many animals the act of walking is evidently acquired by experience; but according to Mr. Bain, even those animals whose "nerves and muscles are more highly matured at the moment of birth," learn all their proper movements of locomotion by experience and practice, and "stumble upon their most needful operations in the course of a

---

* *Emotions and the Will*, p. 355. † *Observations*, Prop. 46.

‡ *Theological and Miscellaneous Works*, vol. iii.; *Remarks on Dr. Reid's Inquiry*, sect. viii.

little time."* Nay, the act of drinking and taking food is acquired only after many tedious trials. "In the course of its rambles and pokings, the young animal encounters a stream and applies its mouth to the surface, putting out the tongue and executing some of those movements of tongue and jaw already associated with the contact of objects of food!" But how, not only by mere *accident*, but by the *same* accident *repeated* a sufficient number of times, can be explained that *particular* association or co-ordination of numerous muscles exactly suited to effect those *particular* movements of tongue and jaw which the animal's wants required? On this assumption, not only the duckling, which takes to the water and swims and walks as soon as it is hatched, must learn to do so by *accidental* associations frequently repeated, but even the humblest creature that crawls, and the meanest insect that flutters on the wing, although they perform those operations perfectly on the first trial, must nevertheless be taught by education and experience; for in the opinion of Mr. Bain, these operations are truly voluntary, and *acquisition*, *education*, and *experience* are the necessary conditions of every act of volition, as opposed to *instinct*. "The overwhelming and fatal course of the moth towards the flame," says Mr. Bain, "is really and truly a volitional impulse."†

With the view of obtaining a clearer insight into the origin and development of voluntary power, let us consider the character of the movements, as well as the mental or psychæal condition, of the new-born infant. Its movements, although spontaneous or automatic, are for the most part definite, but few in kind. They consist chiefly of flexion and extension of the legs, arms, and fingers, with some rotation of the arms and legs; and of the associate movements of the eyes, of crying, and of sucking. The motions of the legs, arms, fingers, and eyes—which are constantly recurring in the intervals between sleep—although they are performed by the child without any purpose or design, are nevertheless the result of certain peculiar feelings, inclinations, or instinctive impulses, which, if restrained in their effects on the muscles, are attended with uneasiness or distress, and by diversion into a new channel, excite the equally automatic actions of crying, which, although undesigned by the infant, are beautifully designed by

---

* *Emotions and the Will*, p. 352.

† *Emotions and the Will*, p. 371. "*Instinct* is defined by being opposed to acquisition, education, and experience." (*The Senses and the Intellect*, chap. iv. p. 256.) Mr. Bain, however, does not altogether deny the existence of *instinctive* feelings and actions; such are reflex actions, the movements of the heart, respiration, &c. The error of Mr. Bain and Dr. Priestley appears to consist in the opinion, that because many associated movements, like those of sucking, can be subsequently performed, and perhaps improved, by voluntary effort, they were therefore originally acquired by *accidental* association and experience.

the Creator as a means of expressing to others the uneasy state of its feelings. In the same way the feeling of uneasiness arising from hunger, which the child itself is unable to gratify, is undesignedly and instinctively made known to those who are able to supply its wants. Now, at this early period of its existence the whole psychæal activity of the infant appears to consist, on the one hand, of a succession of such instinctive feelings, inclinations, or impulses, which are expended in the direct excitement of a variety of muscular movements, as a series of experiments, instituted indeed by nature, but not designed by the child; and on the other hand, of just sufficient intelligence to notice and retain or remember the sequence of events so as to *associate* the effects of its muscular movements with the feelings or impulses which produce them, and store up the knowledge thus acquired for future use, as a basis of its *voluntary* impulses, which gradually increase in number and replace those that are automatic, in proportion as its intellect and special susceptibilities for external objects and impressions become more and more developed. But these primitive feelings or instinctive impulses, which undesignedly and immediately excite contraction in the muscles, *without the intervention of any idea* whatever, correspond, or are similar in nature, to those *subsequent* feelings of inclination or impulse to action, which are excited by the presence of external objects, and which, together with the *idea* of the action to be performed, constitute the "Will" or power of volition. For what is a desire to perform any particular action but a *feeling of inclination or impulse* excited directly or indirectly by an external impression on the senses, and guided to a definite end or rendered intentional by association with the *idea* of the action to be performed? Thus the child, at first, perceives that an object *is secured* by the particular act of *grasping*, although that act resulted from an unintentional or instinctive impulse; and these two facts—the possession of the object and the act which secures it—will become connected in the mind by association. Now if, on a subsequent occasion, the object, when presented, should prove desirable, that is, should have an attraction for the child, by operating through the senses on a special susceptibility, it would excite *an inclination to secure it,* and therefore, by association, an inclination or impulse *to perform the act of grasping* by which it *must be secured.* But the inclination or impulse *now* acting on the muscles, although a more vivid and definite feeling, excited by the object presented, and constituting the *dynamic* element of a desire, will nevertheless be similar in its *nature* and *mode of action* to the inclination or impulse which *originally and spontaneously* performed the same act, *without the idea or design,* and will differ from it only in now exciting the particular act of grasping by

*association* with the *idea* of that action, *as the necessary means of securing the object desired.* A *voluntary impulse,* therefore, is a complex state of consciousness compounded of two elements fused as it were together by association. *In its mode of action* on the muscles it is purely instinctive, and apparently similar to the natural instinct or instinctive intelligence that performs from the first the same kind of operations in the lower animals, or to the emotional impulse in man. Those actions which have already occurred spontaneously, as flexion and extension of the limbs, and flexion of the fingers in grasping, will of course be readily performed by a *voluntary effort,* since the impulses by which they were spontaneously performed were associated at birth with the *necessary muscular co-ordination;* so that nothing more is required than *a knowledge or idea of the movement necessary.* But in proportion as this movement, and consequently the muscular co-ordination on which it depends, differs from that which was *naturally* associated with its spontaneous or instinctive impulse, will be the difficulty of performing it *by a voluntary effort;* for then it will be needful to learn not only *what kind* of action is required, but *how* or *by what effort* it is to be performed; and such knowledge can be acquired only by repeated trial and experience, through which the necessary muscular co-ordination becomes intimately associated with the necessary impulse or effort.

In the new-born infant the faculties of special sense are incomplete, and must await the teachings of time and experience. The hearing is dull, for the cavity of the tympanum contains a fluid which only gradually disappears. Vision is imperfect, the perception of objects being confused and indistinct; for the eyes at first, as may be seen, are not *arrested* by objects, or directed to any particular spot, but move by a constant, unmeaning, irregular and jerking kind of action, from deficient control over their muscles. Moreover, they are sometimes obscured by a kind of film or membrane, and a greater convexity of their globes and lenses limit the vision for some time to things that are near. By degrees, however, the stimulus of light and of bright objects attracts and fixes the eyes, while the feeling of curiosity or a desire to see things more distinctly stimulates the mind to attempt and acquire by practice that control over their movements which is necessary for the proper convergence of their axes, in adaptation to varying distances: so that perhaps the first exertions of voluntary effort are employed in learning some of the acquired perceptions of vision. When objects by this means have become distinct, the same feeling of curiosity impels the infant to reach, handle, and feel them. Its first attempts are more or less unsuccessful, in proportion as the particular movements required differ from those that have already occurred spontaneously, as the result of instinctive or unintentional impulses. The difficulty consists in modifying, when necessary, the originally automatic movements, by restraining the action of some muscles of a group, and co-ordinating the rest with others in a way in which they never spontaneously or automatically acted together. But the *immediate action of the effort on the muscles* is guided by a knowledge or idea of the direction required, and is really instinctive; for the child, of course, is not conscious even of the existence of its muscles. By degrees, however, and under the guidance of the already educated sight, the infant succeeds in correcting its mistakes and in moving its limbs in the directions required. At the same time, it is making progress in other kinds of knowledge, namely, in a further acquaintance with the acquired perceptions of vision, with the notion of extension, and with the physical properties of bodies by the sense of touch combined with the muscular sense. The voluntary execution, therefore, of by far the greater number of particular movements is not learned by a previous *accidental* association of those movements with particular *accidental* sensations; but, as I have endeavoured to explain, by the association of certain efforts or impulses with the requisite muscular co-ordination, discovered on trial, and rendered perfect by repetition. In this way the child endeavours and learns to walk and balance its body; and in after-life, when the same being attempts to learn the varied and complicated movements necessary for certain mechanical operations, he *desires* to perform those movements and persists in the effort to do so, until the necessary associations are acquired.

(*To be continued.*)

---

